# Antecedents of adolescent students’ ICT self-efficacy: The ICT dataset

**DOI:** 10.1016/j.dib.2020.106437

**Published:** 2020-10-22

**Authors:** Xueliang Chen, Jie Hu

**Affiliations:** Department of Linguistics, School of International Studies, Zhejiang University, Hangzhou City 310058, Zhejiang Province, China

**Keywords:** Computer-mediated education, ICT literacy, Interactive learning environments, PISA 2015, Secondary education

## Abstract

Based on the Programme for International Student Assessment (PISA) 2015 dataset, the information and communication technology (ICT) dataset focuses specifically on ICT-related constructs in the context of educational technology. It includes a wide range of student-level variables collected from 30 Economic Co-operation and Development (OECD) countries, which pertain to students’ motivational and behavioural characteristics in relation to their ICT self-efficacy. In total, it comprises 201, 652 students from 7708 schools. As technology has become an integral component of education, the ICT dataset can serve as a handy resource for studying ICT-related constructs. Besides, the ICT dataset holds advantages over the original PISA dataset for its intensive focus and easy readability. With this important resource, researchers can undertake their own research in the neighbouring fields of ICT, developing their own theories or validating existing theoretical frameworks and statements. The focus of this study is to identify the antecedents of adolescent students’ ICT self-efficacy and illuminate potential mechanisms at work.

## Specifications Table

**Table:** 

Subject	Education
Specific subject area	Information and communication technology (ICT), ICT self-efficacy
Type of data	7 Tables (Tables 1–7) 1 Figure (Fig. 1) CSV files 3 R codes Supplementary Materials (Correlation Matrix)
How data were acquired	Data were acquired from the Programme for International Student Assessment (PISA) 2015 dataset (URL: http://www.oecd.org/pisa/data/2015database/). Based on a series of student, teacher, principal and curriculum questionnaires [3], only data aiming to identify ICT-related predictors of students’ performance were acquired from the Student Questionnaire and the ICT Familiarity Questionnaire (URL: http://www.oecd.org/pisa/data/2015database/), which were administered in 30 Organization for Economic Co-operation and Development (OECD) countries and regions around the world.
Data format	Raw; filtered
Parameters for data collection	Aiming to investigate the relationship between adolescents’ interest in the ICT and their ICT self-efficacy, the dataset was collected using eight variables related to ICT interest and ICT self-efficacy in the PISA 2015, which made the dataset unique with an added value in this study. These eight variables included that (1) The independent variable, namely, students’ interest in ICT (coded as INTICT), was developed for the first time in the PISA 2015; (2) The dependent variable, namely, students’ self-perceived competence in using ICT (coded as COMPICT), was also developed for the first time in the PISA 2015; (3) The four mediating variables were categorized as behavioral factors, namely, “use of ICT at school in general” (USESCH), “ICT use outside of school for schoolwork” (HOMESCH), “ICT use outside of school for leisure (ENTUSE)”, and “students’ ICT as a topic in social interaction” (SOIAICT); (4) The two control variables were student gender and the index of economic, social, and cultural status (ESCS).
Description of data collection	The primary data were drawn from the official OECD website (URL: http://www.oecd.org/pisa/data/2015database/) with a series of questionnaires (URL: http://www.oecd.org/pisa/data/2015database/). In this study, the data were collected from the Student Questionnaire and the ICT Familiarity Questionnaire data files with eight variables, which included student-level responses to a wide range of background variables and outcome measures [3]. The raw data were provided in the supplementary materials. In particular, INTICT was derived based on six items that measured students’ overall enjoyment of ICT; COMPICT was derived from five items that measured how competent students perceived themselves to be in using ICT-related knowledge or skills; USESCH, HOMESCH, ENTUSE and SOIAICT were derived from a total of 9, 12, 13, and 5 items, respectively, that emphasized the extent to which students were physically involved in ICT-related activities. Altogether, 30 OECD countries were selected because of their similar cultural and economic status.
Data source location	Global Primary data sources: PISA 2015 dataset from OECD (URL: http://www.oecd.org/pisa/data/2015database/)
Data accessibility	With the article
Related research article	Authors’ names: Xueliang Chen, Jie Hu Title: ICT-related behavioral factors mediate the relationship between adolescents’ ICT interest and their ICT self-efficacy: Evidence from 30 countries Journal: Computers & Education Reference: CAE 104004 Article reference: CAE_CAE-D-19–01964 https://doi.org/10.1016/j.compedu.2020.104004

## Value of the Data

• The ICT dataset distinguishes itself though a narrow but intensive focus on ICT-related constructs, bearing relevance to today's educational reality.
• The dataset could facilitate an understanding of the complex relationships between ICT-related motivational and behavioural factors.
• The ICT dataset holds advantages over the original PISA dataset for its easy accessibility and simple structure.

**Fig. 1:**
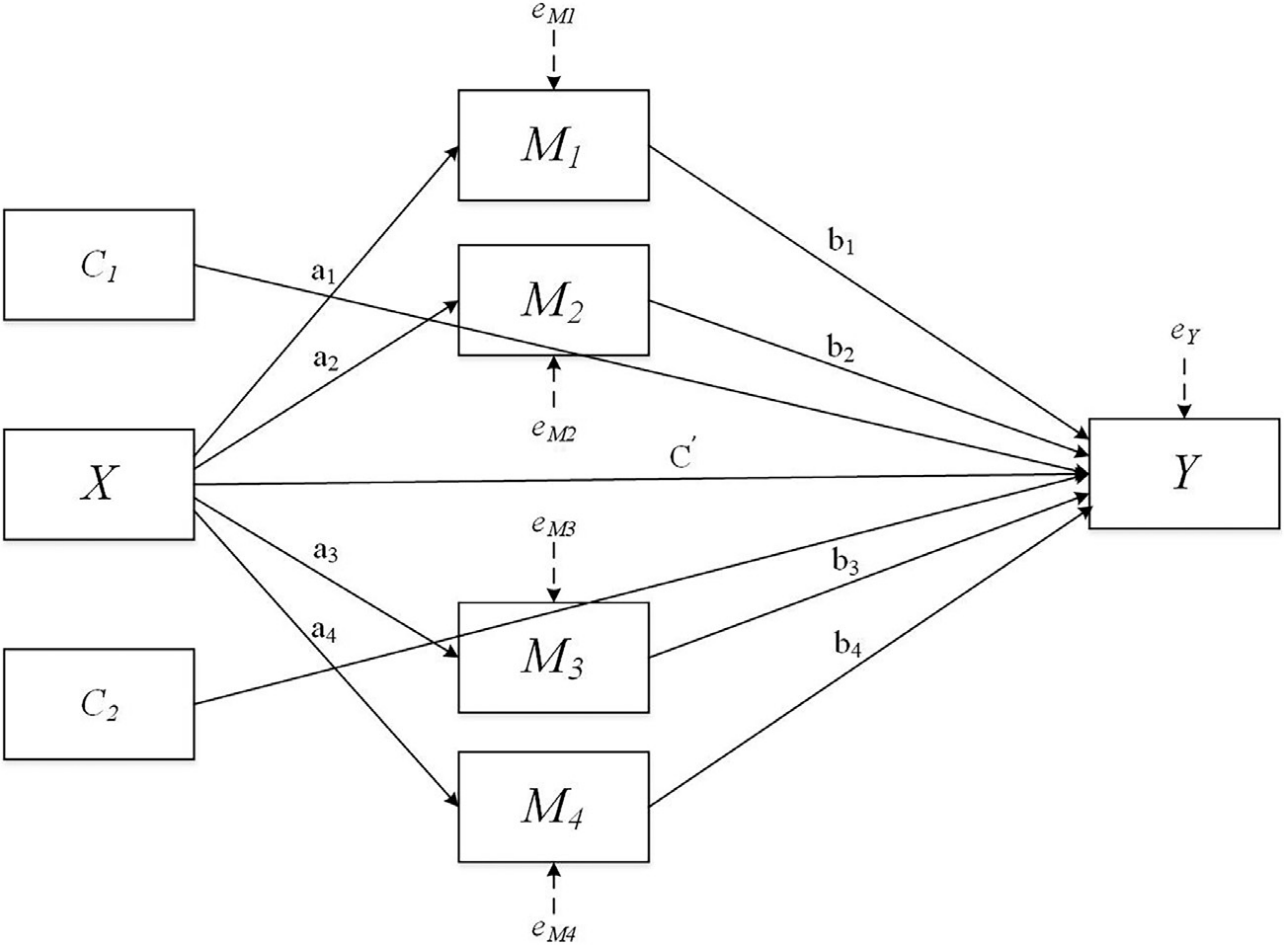
Statistical diagram representing the parallel multiple mediator model with 4 mediators.

Note. X: Interest in ICT; Y: Perceived ICT competence at age fifteen; M1: ICT use at school; M2: ICT use outside of school for schoolwork; M3: ICT use outside of school for leisure; M4: ICT use in social interaction; C1: Student gender; C2: ESCS.

## 1 Data Description

To investigate the relationship between adolescents’ interest in information and communication technology (ICT) and their ICT self-efficacy, this dataset was compiled using a number of ICT-related variables from the Programme for International Student Assessment (PISA) 2015 dataset (URL: http://www.oecd.org/pisa/data/2015database/) using the programming language R (URL: https://www.R-project.org), resulting in a sample of 201,652 students from 7708 schools from 30 Economic Co-operation and Development (OECD) countries. During data pre-processing, missing data were filtered with the expectation-maximization (EM) algorithm [1] using the statistical package SPSS 20. A 1-1-1 multilevel mediation model was adopted for data analysis, which was performed using the *lavaan* package [5] in R. The independent variable was adolescent students’ ICT interest. The dependent variable was ICT self-efficacy. The mediators included students’ ICT use at school, outside of school for homework and leisure, and ICT use for social interaction. The control variables included student gender and socioeconomic status. Given the complexity and gigantic volume of the original dataset, this accessible dataset would serve as a unique and much-needed replacement for researchers who are interested in ICT-related constructs.

All the following tables are from the related research article [4].

Information relating to the sample and the descriptive statistics of the main variables was provided in Table 1 for each individual country.

**Table 1:** Statistical information of the samples.

Country	*N* (students)	Male%	N (schools)	INTICT		COMPICT	
				M	SD	M	SD
Australia	14,530	51	758	0.1444	0.8695	0.1821	0.8333
Austria	7007	51	269	0.0645	0.9870	−0.0876	1.0191
Belgium	9651	51	288	0.1132	0.9490	0.0331	0.8955
Chile	7053	50	227	0.0817	0.9261	0.0991	0.9087
Czech	6894	50	344	−0.1592	0.9002	−0.1219	0.9223
Denmark	7161	50	333	0.1775	0.8566	0.2170	0.8844
Estonia	5587	50	206	−0.1324	0.8602	−0.0393	0.9214
Finland	5882	51	168	−0.1137	0.8828	−0.0903	0.8729
France	6108	49	252	0.3115	1.0465	0.2315	0.9724
Germany	6504	51	256	0.0463	0.8826	−0.0584	0.9360
Greece	5532	51	211	0.1280	0.9280	0.0585	0.9154
Hungary	5658	50	245	−0.2646	0.9197	0.0806	0.9471
Iceland	3371	48	124	0.0527	0.9371	−0.0079	0.8869
Ireland	5741	51	167	0.3194	0.8688	0.2065	0.8862
Israel	6598	44	173	−0.0457	1.0579	−0.0175	0.9817
Italy	11,583	50	474	−0.1056	0.8866	−0.0742	0.882
Japan	6647	50	198	−0.4468	1.1112	−0.9456	1.0076
Korea	5581	52	168	−0.3658	0.9118	−0.5677	0.8873
Latvia	4869	50	250	−0.1861	0.8859	−0.1209	0.8823
Luxembourg	5299	49	44	0.0010	0.9996	0.0029	0.9915
Mexico	7568	50	275	−0.1739	0.9727	−0.0660	1.0186
Netherlands	5385	50	187	0.0546	0.8796	−0.0393	0.8357
Zealand	4520	50	183	0.1833	0.8684	0.2045	0.8147
Poland	4478	51	169	−0.1440	0.8724	0.0116	0.9207
Portugal	7325	50	246	0.3602	1.0026	0.3401	0.8951
Slovak	6350	52	290	−0.2591	0.9171	−0.1331	0.9271
Slovenia	6406	55	333	−0.1264	0.9403	0.0502	0.9625
Sweden	5458	50	202	0.2305	0.9628	0.2605	0.9509
Switzerland	5860	52	227	−0.0455	0.9391	0.0103	0.9721
UK	11,046	51	441	0.2985	0.6592	0.3500	0.6383

**Table 2:** Scale reliabilities for ICT familiarity questionnaire indices in 30 OECD countries.

Country	INTICT	COMPICT	HOMESCH	ENTUSE	USESCH	SOIAICT
Australia	0.7850	0.8480	0.9060	0.8040	0.8360	0.8500
Austria	0.7650	0.8400	0.8850	0.7840	0.8570	0.8640
Belgium	0.7940	0.8460	0.9190	0.7970	0.9100	0.8550
Chile	0.7970	0.8390	0.9110	0.8310	0.8670	0.8590
Czech Republic	0.7750	0.8580	0.9010	0.8100	0.8870	0.8800
Denmark	0.7370	0.851	0.8600	0.7920	0.7690	0.8430
Estonia	0.7820	0.8460	0.8850	0.7790	0.8990	0.8680
Finland	0.7920	0.8520	0.9160	0.8010	0.8510	0.8510
France	0.8180	0.8620	0.9170	0.8200	0.8890	0.8590
Germany	0.7550	0.8410	0.8540	0.8340	0.8430	0.8020
Greece	0.7710	0.8310	0.9330	0.8500	0.9300	0.8510
Hungary	0.7780	0.8720	0.9290	0.8230	0.9120	0.8780
Iceland	0.8090	0.8320	0.9190	0.7860	0.8670	0.8430
Ireland	0.7370	0.8200	0.8870	0.7880	0.8510	0.8490
Israel	0.8490	0.8850	0.9380	0.8720	0.9380	0.9040
Italy	0.7530	0.8270	0.9140	0.8120	0.8860	0.8140
Japan	0.8560	0.8750	0.8400	0.7790	0.7850	0.8880
Korea	0.8240	0.8540	0.9060	0.7770	0.9270	0.8830
Latvia	0.7760	0.8210	0.9020	0.8070	0.8870	0.7950
Luxembourg	0.8000	0.8570	0.9220	0.8150	0.9090	0.8830
Mexico	0.8270	0.8800	0.9160	0.8890	0.9010	0.8400
Netherlands	0.7490	0.8220	0.8490	0.7360	0.8270	0.8390
New Zealand	0.7890	0.8390	0.9200	0.8060	0.8730	0.8420
Poland	0.7440	0.8660	0.8900	0.8120	0.9030	0.8370
Portugal	0.8060	0.8660	0.9430	0.8500	0.9110	0.8590
Slovak Republic	0.8010	0.8670	0.9230	0.8400	0.9030	0.8430
Slovenia	0.7720	0.8680	0.8960	0.8080	0.9070	0.8430
Sweden	0.8110	0.8760	0.9280	0.8050	0.8780	0.9020
Switzerland	0.7550	0.8460	0.9030	0.7990	0.8790	0.8590
UK	0.7620	0.8400	0.9010	0.7870	0.8390	0.8460

**Table 3:** ICC for each of the 30 OECD countries.

Country	Between-school variance	Within-school variance	Total variance	ICC
Australia	0.0066	0.6878	0.6944	0.0095
Austria	0.0434	0.9945	1.0379	0.0418
Belgium	0.0136	0.7881	0.8017	0.0169
Chile	0.0223	0.8035	0.8258	0.0270
Czech Republic	0.0223	0.8035	0.8258	0.0270
Denmark	0.0083	0.7739	0.7822	0.0107
Estonia	0.0021	0.8469	0.8490	0.0025
Finland	0.0064	0.7557	0.7620	0.0083
France	0.0013	0.9443	0.9456	0.0014
Germany	0.0029	0.8733	0.8762	0.0033
Greece	0.0050	0.8330	0.8380	0.0060
Hungary	0.0183	0.8790	0.8973	0.0204
Iceland	0.0067	0.7800	0.7866	0.0085
Ireland	0.0093	0.7760	0.7854	0.0119
Israel	0.0463	0.9173	0.9636	0.0481
Italy	0.0242	0.7533	0.7776	0.0312
Japan	0.0162	0.9991	1.0154	0.0160
Korea	0.0154	0.7720	0.7874	0.0196
Latvia	0.0044	0.7741	0.7785	0.0057
Luxembourg	0.0119	0.9723	0.9842	0.0121
Mexico	0.0915	0.9512	1.0427	0.0877
Netherlands	0.0037	0.6947	0.6983	0.0053
New Zealand	0.0037	0.6947	0.6983	0.0053
Poland	0.0101	0.8376	0.8477	0.0119
Portugal	0.0112	0.7900	0.8013	0.0140
Slovak Republic	0.0112	0.7900	0.8013	0.0140
Slovenia	0.0436	0.8823	0.9258	0.0470
Sweden	0.0081	0.8961	0.9042	0.0090
Switzerland	0.0243	0.9212	0.9454	0.0257
UK	0.0042	0.4032	0.4075	0.0103

*Note*. The intraclass correlation coefficient is calculated as the proportion of total variance that is accounted for by the clustering of students in schools.

**Table 4:** Relative total effects of ICT interest on ICT self-efficacy.

Country	B (95% CI)	Beta	BootSE
Australia	0.5040 (0.4876, 0.5206)	0.5279	0.0087
Austria	0.4938 (0.4672, 0.5157)	0.4815	0.0122
Belgium	0.4931 (0.4710, 0.5137)	0.5254	0.0109
Chile	0.5099 (0.4830, 0.5359)	0.5213	0.0132
Czech Republic	0.5259 (0.4978, 0.5534)	0.5161	0.0145
Denmark	0.4908 (0.4610, 0.5161)	0.4785	0.0141
Estonia	0.4843 (0.44892, 0.5157)	0.4539	0.0163
Finland	0.4878 (0.4610, 0.5187)	0.4952	0.0146
France	0.4743 (0.4507, 0.4965)	0.5138	0.0117
Germany	0.5261 (0.5037, 0.5529)	0.4974	0.0129
Greece	0.4890 (0.4600, 0.5178)	0.4978	0.0153
Hungary	0.5276 (0.5002, 0.5559)	0.5126	0.0143
Iceland	0.4706 (0.4375, 0.5082)	0.4995	0.0185
Ireland	0.4795 (0.4524, 0.5099)	0.4717	0.0142
Israel	0.5425 (0.5178, 0.5643)	0.5849	0.0117
Italy	0.5166 (0.4953, 0.5358)	0.5218	0.0102
Japan	0.5703 (0.5475, 0.5895)	0.6335	0.0106
Korea	0.4529 (0.4228, 0.4849)	0.4706	0.0159
Latvia	0.4377 (0.4056, 0.4734)	0.4385	0.0169
Luxembourg	0.5256 (0.4943, 0.5515)	0.5328	0.0147
Mexico	0.4883 (0.4617, 0.5139)	0.4694	0.0139
Netherlands	0.4469 (0.4189, 0.4739)	0.4728	0.0145
New Zealand	0.4893 (0.4589, 0.5194)	0.5229	0.0155
Poland	0.4806 (0.4451, 0.5160)	0.4554	0.0181
Portugal	0.4604 (0.4395, 0.4825)	0.5173	0.0110
Slovak Republic	0.5028 (0.4720, 0.5289)	0.4989	0.0146
Slovenia	0.5194 (0.4905, 0.5498)	0.5079	0.0149
Sweden	0.4736 (0.4391, 0.5018)	0.4798	0.0153
Switzerland	0.5073 (0.4797, 0.5347)	0.4914	0.0143
UK	0.4769 (0.4520, 0.5013)	0.4938	0.0129

*Note*. B: unstandardized model coefficient. 95% CI = 95% bias-corrected confidence intervals based on the bootstrapping method. 95% confidence intervals that do not contain zero indicate significant results. Beta: standardized model coefficient. Bootstrapping is based on 1000 samples.

**Table 5:** Effect of the control variables: student gender and ESCS.

Country	Student gender		ESCS	
	B (95% CI)	BootSE	B (95% CI)	BootSE
Australia	−0.1896 (−0.2134, −0.1668)	0.0117	0.0650 (0.0498, 0.0784)	0.0074
Austria	−0.3842 (−0.4265, −0.3451)	0.0202	0.0923 (0.0666, 0.1151)	0.0121
Belgium	−0.2537 (−0.2832, −0.2216)	0.0156	0.0246 (0.0083, 0.0413)	0.0086
Chile	−0.1996 (−0.2385, −0.1661)	0.0178	0.0514 (0.0355, 0.0673)	0.0081
Czech Republic	−0.3648 (−0.4006, −0.3315)	0.0174	0.0593 (0.0361, 0.0836)	0.0121
Denmark	−0.3171 (−0.3479, −0.2819)	0.0167	0.0454 (0.0286, 0.0659)	0.0094
Estonia	−0.3372 (−0.3805, −0.2942)	0.0216	0.1130 (0.0861, 0.1406)	0.0141
Finland	−0.3999 (−0.4380, −0.3634)	0.0190	0.0515 (0.0283, 0.0801)	0.0129
France	−0.2903 (−0.3289, −0.2468)	0.0217	0.0710 (0.0454, 0.0961)	0.0131
Germany	−0.4369 (−0.4713, −0.3936)	0.0197	0.0352 (0.0131, 0.0604)	0.0117
Greece	−0.2271 (−0.2661, −0.1855)	0.0208	0.0827 (0.0616, 0.1074)	0.0111
Hungary	−0.1962 (−0.2366, −0.1534)	0.0221	0.0992 (0.0761, 0.1207)	0.0115
Iceland	−0.2341 (−0.2882, −0.1862)	0.0269	0.0241 (−0.0112, 0.0586)	0.0179
Ireland	−0.2176 (−0.2578, −0.1754)	0.0205	0.0518 (0.0292, 0.0768)	0.0118
Israel	−0.2227 (−0.2617, −0.1855)	0.0197	0.1024 (0.0780, 0.1257)	0.0121
Italy	−0.2386 (−0.2650, −0.2119)	0.0138	0.0422 (0.0261, 0.0566)	0.0079
Japan	−0.2549 (−0.2913, −0.2179)	0.0190	0.0372 (0.0106, 0.0621)	0.0132
Korea	−0.2321 (−0.2754, −0.1902)	0.0215	0.1450 (0.1132, 0.1761)	0.0160
Latvia	−0.3336 (−0.3763, −0.2900)	0.0222	0.0972 (0.0703, 0.1215)	0.0129
Luxembourg	−0.3408 (−0.3833, −0.2956)	0.0224	0.0706 (0.0524, 0.0905)	0.0010
Mexico	−0.2045 (−0.2418, −0.1658)	0.0188	0.1678 (0.1490, 0.1840)	0.0088
Netherlands	−0.3044 (−0.3444, −0.2674)	0.0193	0.0383 (0.0117, 0.0656)	0.0135
New Zealand	−0.1610 (−0.2016, −0.1173)	0.0211	0.0869 (0.0578, 0.1142)	0.0143
Poland	−0.3310 (−0.3769, −0.2844)	0.0233	0.1292 (0.0938, 0.1548)	0.0148
Portugal	−0.2729 (−0.3082, −0.2389)	0.0179	0.0664 (0.0509, 0.0812)	0.0078
Slovak Republic	−0.2435 (−0.2842, −0.2059)	0.0196	0.1193(0.0988, 0.1397)	0.0103
Slovenia	−0.3241 (−0.3634, −0.2824)	0.0208	0.0473 (0.0221, 0.0713)	0.0125
Sweden	−0.3198 (−0.3618, −0.2800)	0.0213	0.0956 (0.0686, 0.1224)	0.0140
Switzerland	−0.4116 (−0.4507, −0.3709)	0.0210	0.0389 (0.0184, 0.0632)	0.0114
UK	−0.1236 (−0.1433, −0.1039)	0.0103	0.0825 (0.0689, 0.0949)	0.0065

*Note*. B: unstandardized model coefficient. 95% CI = 95% bias-corrected confidence intervals based on the bootstrapping method. 95% confidence intervals that do not contain zero indicate statistically significant results. Bootstrapping is based on 1000 samples.

**Table 6:** Relative direct effects of ICT interest on ICT self-efficacy.

Country	B (95% CI)	Beta	BootSE
Australia	0.3707 (0.3511, 0.3902)	0.3884	0.0100
Austria	0.3507 (0.3230, 0.3739)	0.3419	0.0131
Belgium	0.3599 (0.3334, 0.3820)	0.3834	0.0119
Chile	0.3936 (0.3632, 0.4228)	0.4023	0.0150
Czech Republic	0.3571 (0.3287, 0.3912)	0.3504	0.0154
Denmark	0.3581 (0.3254, 0.3891)	0.3491	0.0158
Estonia	0.3202 (0.2830, 0.3539)	0.3001	0.0176
Finland	0.3171 (0.2860, 0.3484)	0.3219	0.0157
France	0.3276 (0.2993, 0.3547)	0.3548	0.0142
Germany	0.3717 (0.3460, 0.4004)	0.3513	0.0141
Greece	0.3308 (0.2995, 0.3658)	0.3368	0.0168
Hungary	0.3892 (0.3538, 0.4200)	0.3781	0.0172
Iceland	0.3249 (0.2831, 0.3660)	0.3449	0.0210
Ireland	0.3443 (0.3151, 0.3740)	0.3387	0.0152
Israel	0.3871 (0.3611, 0.4133)	0.4173	0.0136
Italy	0.3518 (0.3287, 0.3743)	0.3554	0.0113
Japan	0.3413 (0.3147, 0.3660)	0.3791	0.0130
Korea	0.2750 (0.2453, 0.3070)	0.2857	0.0160
Latvia	0.3105 (0.2760, 0.3466)	0.3111	0.0179
Luxembourg	0.3706 (0.3349, 0.4042)	0.3757	0.0171
Mexico	0.3442 (0.3134, 0.3734)	0.3308	0.0152
Netherlands	0.3486 (0.3188, 0.3768)	0.3688	0.0150
New Zealand	0.3732 (0.3406, 0.4092)	0.3989	0.0173
Poland	0.3156(0.2756, 0.3516)	0.2990	0.0195
Portugal	0.3283 (0.3041, 0.3519)	0.3690	0.0123
Slovak Republic	0.3482 (0.3145, 0.3784)	0.3456	0.0168
Slovenia	0.3503 (0.3155, 0.3835)	0.3426	0.0173
Sweden	0.3400 (0.3019, 0.3692)	0.3445	0.0165
Switzerland	0.3734 (0.3407, 0.4033)	0.3617	0.0162
UK	0.3853 (0.3585, 0.4141)	0.3989	0.0145

*Note*. B: unstandardized model coefficient. 95% CI = 95% bias-corrected confidence intervals based on the bootstrapping method. 95% confidence intervals that do not contain zero indicate statistically significant results. Beta: standardized model coefficient. Bootstrapping is based on 1000 samples.

**Table 7:** Relative indirect effects of ICT interest on ICT self-efficacy and the proportion mediated.

	M1 (HOMESCH)		M2 (ENTUSE)		M3 (USESCH)		M4 (SOIASCH)		
Country	a_1_b_1_ (95% CI)	BootSE	a_2_b_2_ (95% CI)	BootSE	a_3_b_3_ (95% CI)	BootSE	a_4_b_4_ (95% CI)	BootSE	Proportion##### Mediated
Australia	**−0.0069#####(−0.0115, 0.0025)**	0.0023	0.0390#####(0.0298, 0.0481)	0.0046	**0.0044#####(−0.0010, 0.0099)**	0.0028	0.0967#####(0.0876, 0.1075)	0.0049	0.2643
Austria	**0.0013#####(−0.0030, 0.0063)**	0.0023	0.0389#####(0.0294,0.0500)	0.0052	**−0.0034#####(−0.0095,0.0031)**	0.0033	0.1062#####(0.0933, 0.1195)	0.0066	0.2896
Belgium	**−0.0020#####(−0.0061, 0.0018)**	0.0019	0.0413#####(0.0320,0.0504)	0.0046	**−0.0005#####(−0.0035, 0.0023)**	0.0015	0.0945#####(0.0831, 0.1053)	0.0055	0.2703
Chile	**−0.0045#####(−0.0095, 0.0004)**	0.0025	0.0315#####(0.0234, 0.0412)	0.0046	**0.0023#####(−0.0019, 0.0066)**	0.0022	0.0870#####(0.0757, 0.1009)	0.0064	0.2281
Czech Republic	**−0.0014#####(−0.0094, 0.0065)**	0.0041	0.0391#####(0.0284, 0.0507)	0.0058	**0.0004#####(−0.0060, 0.0078)**	0.0034	0.1307#####(0.1154, 0.1456)	0.0078	0.3210
Denmark	**−0.0016#####(−0.0100,0.0065)**	0.0042	0.0432#####(0.0306,0.0538)	0.0059	**−0.0008#####(−0.0104, 0.0078)**	0.0045	0.0920#####(0.0804, 0.1048)	0.0064	0.2706
Estonia	**−0.0038#####(−0.0116, 0.0026)**	0.0035	0.0259#####(0.0147, 0.0389)	0.0062	**0.0005#####(−0.0049, 0.0064)**	0.0030	0.1415#####(0.1244, 0.1608)	0.0093	0.3388
Finland	−0.0055#####(−0.0115, −0.0008)	0.0027	0.0356#####(0.0204, 0.0502)	0.0073	**0.0028#####(−0.0044, 0.0099)**	0.0036	0.1379#####(0.1208, 0.1559)	0.0089	0.3501
France	0.0070#####(0.0016, 0.0125)	0.0028	0.0350#####(0.0235, 0.0472)	0.0062	−0.0036#####(−0.0082, −0.0002)	0.0021	0.1084#####(0.0942, 0.1238)	0.0072	0.3095
Germany	−0.0047#####(−0.0097, −0.0002)	0.0024	0.0470#####(0.0354, 0.0588)	0.0060	**−0.0031#####(−0.0072, 0.0006)**	0.0020	0.1154#####(0.1002, 0.1296)	0.0073	0.2939
Greece	**0.0015#####(−0.0043, 0.0079)**	0.0032	0.0363#####(0.0243, 0.0486)	0.0061	−0.0023#####(−0.0053, −0.0006)	0.0012	0.1228#####(0.1063, 0.1388)	0.0084	0.3237
Hungary	**−0.0090#####(−0.0177, −0.0016)**	0.0040	0.0385#####(0.0253, 0.0525)	0.0071	**−0.0043#####(−0.0110, 0.0042)**	0.0038	0.1132#####(0.0977, 0.1317)	0.0086	0.2623
Iceland	**−0.0030#####(−0.0129, 0.0051)**	0.0046	0.0390#####(0.0220, 0.0564)	0.0087	**0.0049#####(−0.0037, 0.0133)**	0.0044	0.1047#####(0.0842, 0.1247)	0.0100	0.3094
Ireland	**−0.0024#####(−0.0063, 0.0006)**	0.0018	0.0547#####(0.0431, 0.0670)	0.0062	−0.0042#####(−0.0075, −0.0016)	0.0015	0.0871#####(0.0753, 0.1004)	0.0064	0.2820
Israel	**−0.0123#####(−0.0222, −0.0044)**	0.0046	0.0472#####(0.0357, 0.0597)	0.0062	**−0.0021#####(−0.0075, 0.0029)**	0.0027	0.1226#####(0.1075, 0.1373)	0.0075	0.2865
Italy	**−0.0033#####(−0.0069, 0.0003)**	0.0018	0.0486#####(0.039, 0.0581)	0.0050	**0.0005#####(−0.0030, 0.0039)**	0.0018	0.1190#####(0.1070,0.1299)	0.0057	0.3190
Japan	0.0052#####(0.0001, 0.0098)	0.0025	0.0500#####(0.0387, 0.0614)	0.00609	**−0.0013#####(−0.0047, 0.0022)**	0.0018	0.1751#####(0.1589, 0.1907)	0.0082	0.4015
Korea	**0.0036#####(−0.0008, 0.0090)**	0.0024	0.0335#####(0.0230, 0.0450)	0.0057	**0.0006#####(−0.0010, 0.0024)**	0.0008	0.1401#####(0.1261, 0.1581)	0.00806	0.3926
Latvia	**−0.0089#####(−0.0161, −0.0040)**	0.0031	0.0280#####(0.0182, 0.0408)	0.0056	**−0.0030#####(−0.0085, 0.0014)**	0.0025	0.1112#####(0.093, 0.1293)	0.00893	0.2908
Luxembourg	**−0.0054#####(−0.0131, 0.0010)**	0.0036	0.0448#####(0.0326, 0.0590)	0.0068	**0.0019#####(−0.0030, 0.0073)**	0.0026	0.1137#####(0.0970, 0.1310)	0.00846	0.2949
Mexico	**0.0035#####(−0.0032, 0.0100)**	0.0034	0.0424#####(0.0337, 0.0539)	0.0051	**0.0003#####(−0.0029, 0.0035)**	0.0017	0.0979#####(0.0859, 0.1111)	0.00648	0.2951
Netherlands	**−0.0038#####(−0.0102, 0.0015)**	0.0029	0.0368#####(0.0247, 0.0487)	0.0064	**0.0015#####(−0.0047, 0.008)**	0.0032	0.0638#####(0.0540, 0.0751)	0.0054	0.2200
New Zealand	**0.0046#####(−0.0121, 0.0035)**	0.0040	0.0256#####(0.0119, 0.0392)	0.0069	**0.0001#####(−0.0084, 0.0079)**	0.0043	0.0952#####(0.0799, 0.1137)	0.0082	0.2565
Poland	**−0.0086#####(−0.0174, 0.0021)**	0.0039	0.0357#####(0.0210, 0.0520)	0.0076	**−0.0060#####(−0.0133, 0.0003)**	0.0032	0.1440#####(0.1239, 0.1646)	0.0109	0.3435
Portugal	**−0.0003#####(−0.0042, 0.0040)**	0.0021	0.0360#####(0.0261, 0.0458)	0.0049	−0.0050#####(−0.0091, −0.0010)	0.0021	0.1013#####(0.0901, 0.1142)	0.0059	0.2867
Slovak Republic	**−0.0069#####(−0.0170, 0.0014)**	0.0046	0.0338#####(0.0215, 0.051)	0.0072	**0.0056#####(−0.0026, 0.0141)**	0.0042	0.1220#####(0.1051, 0.1404)	0.0088	0.3073
Slovenia	**−0.007#####(−0.0152, 0.0022)**	0.00434	0.0534#####(0.0391, 0.0674)	0.0073	−0.0071#####(−0.0137, −0.0001)	0.0036	0.1298#####(0.1153, 0.1475)	0.0084	0.3256
Sweden	**−0.0056#####(−0.0143, 0.0016)**	0.00395	**0.0054#####(−0.007, 0.0181)**	0.0061	**0.0059#####(−0.0031, 0.0166)**	0.0048	0.1279#####(0.1116, 0.1479)	0.0087	0.2821
Switzerland	**−0.0088#####(−0.0150, −0.0039)**	0.00283	0.0456#####(0.0317, 0.0588)	0.0069	**0.0029#####(−0.002, 0.0070)**	0.0021	0.0943#####(0.0819, 0.1094)	0.0068	0.2641
UK	**0.0020#####(−0.0017, 0.0059)**	0.0020	0.0175#####(0.0053, 0.0282)	0.0057	−0.0050#####(−0.0104, −0.0008)	0.0024	0.0773#####(0.0649, 0.0897)	0.0063	0.1925

*Note*. 95% CI = 95% bias-corrected confidence intervals based on the bootstrapping method. Confidence intervals that contain zero are deemed nonsignificant and highlighted in bold. Bootstrapping is based on 1000 samples.

The questionnaires from which the variables were derived can be accessed at the website of the OECD (URL: http://www.oecd.org/pisa/data/2015database/), and the technical details of the scale construction and validation procedures can be found in the official 2015 PISA technical report [3].

The eight variables that were used for each OECD country are listed as follows:

**Table:** 

Variable Code	Explanation
INTICT	Interest in ICT
COMPICT	Perceived ICT competence at fifteen
USESCH	ICT use at school
HOMEUSE	ICT use outside of school for school purposes
ENTUSE	ICT use outside of school for leisure
SOIAICT	ICT use in social interaction
ESCS	The index of economic, social, and cultural status
ST004D01T	Student gender

The raw data include information about 201, 652 students from 7708 schools. A total of eight variables were included in the data, including ICT interest, ICT self-efficacy, ICT use at school, ICT use at home for schoolwork, ICT use for leisure, ICT use for social interaction, student gender, and socioeconomic status.

The supplementary materials include the R code for analysis, the correlation matrix for each country, and the raw and imputed data. There are three R scripts in the supplementary materials, one for the calculation of the intraclass correlation coefficient (ICC), one for the calculation of the regression coefficients of the control variables, including student gender and socioeconomic status (ESCS), and one for multi-level mediation analysis. These three scripts are coded as “R code for ICC”, “R code for the control variables”, and “R code for multilevel mediation”, respectively, all of which can be found in the supplementary materials. The correlation matrix includes the bivariate correlations among all the variables in the imputed dataset, which are calculated individually simultaneously for each OECD country. The raw data are a subset of the PISA 2015 student dataset, which contains only the ICT-related constructs, whereas the imputed data have been imputed using the expectation-maximization algorithm.

## 2 Experimental Design, Materials and Methods

Based on the PISA 2015 dataset, 30 OECD countries were selected for analysis, resulting in a sample of 201, 652 students from 7708 schools. During data pre-processing, missing data were imputed with the expectation-maximization (EM) algorithm [1] using the statistical package SPSS 20. A 1-1-1 multilevel mediation model was adopted for data analysis, which was performed using the *lavaan* package [5] in R (R Core Team, 2019).

To obtain the original data, the official website of the OECD (URL: http://www.oecd.org/pisa/data/2015database/) was accessed, where all the datasets pertaining to the Programme for International Student Assessment (PISA) 2015 were hosted. For the purposes of this study, only the student questionnaire data file (coded as: PUF_SPSS_COMBINED_CMB_STU_QQQ.zip) was downloaded for use. As this dataset contains a large number of variables, only the ICT-related variables used in this study were retained, and the original SPSS format was kept unchanged.

After these variables were selected, they were inspected using the “Analyze>descriptive Statistics>Descriptives” function in SPSS to identify the percentage and patterns of missing data. Then expectation-maximization was performed on these original variables to impute missing values using the “EM” function contained in “Missing Value Analysis”. These pre-processing steps resulted in the final data used in our original study, which contains variables as mentioned below.

In accordance with the research questions, two variables were used as control variables (student gender, socioeconomic status), one was used as the independent variable (adolescents’ ICT interest), four were selected as the mediating variables (ICT at home, ICT use at school, ICT use for leisure, ICT use for social interaction), and one as the outcome variable (adolescents’ ICT self-efficacy at age fifteen). Given that educational data are inherently hierarchical, this structure needs to be taken into account as well. Therefore, a 1-1-1 multilevel mediation model was constructed to investigate the research questions. This analysis starts with a calculation of the intraclass correlation coefficient (ICC), which was used to gauge the magnitude of the clustering effect caused by the data structure. This was performed using the *lmer* package [2] in R. Then, the main analysis was performed using the *lavaan* package [5], which can be used to account for the hierarchical structure of the data along with the mediation analysis. During this process, the regression coefficients for the control and main variables were returned, along with their confidence intervals and standard errors.

## Declaration of Competing Interest

The authors declare that they have no known competing financial interests or personal relationships which have, or could be perceived to have, influenced the work reported in this article.

## Appendix Supplementary materials

Image, application 1

Image, application 2

Image, application 3

Image, application 4

Image, application 5

## References

[1] Dempster A.P., Laird N.M., Rubin D.B. (1977). Maximum likelihood from incomplete data via the EM algorithm. J. R. Stat. Soc..

[2] Bates D., Mächler M., Bolker B., Walker S. (2015). Fitting linear mixed-effects models using *lme4*. J. Stat. Softw..

[3] OECD (2017). PISA 2015 Technical Report.

[4] Chen X.L., Hu J. (2020). ICT-related behavioral factors mediate the relationship between adolescents’ ICT interest and their ICT self-efficacy: evidence from 30 countries. Comput. Educ..

[5] Rosseel Y. (2012). *lavaan*: an R package for structural equation modeling. J. Stat. Softw..

